# Modeling hospital catchment areas in pediatric oncology using an empirically parameterized extended Huff-model

**DOI:** 10.1186/s12942-026-00478-2

**Published:** 2026-06-06

**Authors:** Jonas Kapitza, Thomas Wieland, Markus Metzler

**Affiliations:** 1 Comprehensive Cancer Center Erlangen-EMN, CCC WERA, Erlangen, Germany; 2 Comprehensive Cancer Center Mainfranken, CCC WERA, University Hospital Würzburg, 97080 Würzburg, Germany; 3 Independent researcher, Freiburg, Germany; 4 Pediatric Oncology and Hematology, Department of Pediatrics and Adolescent Medicine, University Hospital Erlangen, Erlangen, Germany

**Keywords:** Hospital catchment areas, Pediatric oncology, Patient mobility, Medical geography, Health services research, Huff model, Market area models

## Abstract

**Background:**

Specialized pediatric oncology is typically concentrated in a few high-volume centers, creating tensions between the need for centralization and equitable spatial access. For regional health planning, robust methods are required to delineate hospital catchment areas and understand how structural site characteristics and accessibility shape patient-to-hospital travel flows. This study uses pediatric oncology in Bavaria, Germany, as a case to develop and test an extended, empirically calibrated Huff model for modeling hospital catchment areas.

**Methods:**

We analyzed 3,320 incident cases of pediatric oncology recorded in the German Childhood Cancer Registry between 2014 and 2023, which were treated at the seven specialized hospitals in Bavaria. An extended Huff model was specified that integrates structural indicators of hospital capacity and quality (bed capacity, staffing, cancer center accreditation), a spatial clustering variable that captures proximity-related interactions among nearby hospital sites, and a logistic distance-decay function based on travel times. Model parameters were estimated using maximum likelihood, and competing specifications were compared primarily using mean absolute percentage error (MAPE). A scenario analysis was conducted to assess how a reduction of nurse staffing ratios at two Munich hospitals would affect patient-to-hospital travel flows and catchment areas.

**Results:**

Our final baseline model, comprising four structural indicators, a clustering variable, and a logistic travel-time function, achieved a MAPE of 5.85% and an R² of 0.89. Capacity and quality indicators displayed positive effects on hospital choice, whereas the clustering parameter was negative, indicating proximity-related interaction effects among nearby hospitals. In the case scenario, a 20% reduction in the nursing staff ratio at the Munich sites led to declining modeled patient shares at both hospitals (− 2.0 and − 2.5% points, respectively) and corresponding gains primarily at Augsburg (+ 3.5% points) and Regensburg (+ 1.3% points), particularly in overlapping and transitional catchment zones.

**Conclusions:**

Our extended Huff model, which combines multidimensional structural indicators, spatial clustering, and realistic travel-time effects, can accurately represent hospital catchment areas and patient-to-hospital travel flows in specialized pediatric oncology. The approach provides a transparent, empirically grounded framework for assessing accessibility, identifying spatial interdependencies between hospital sites, and conducting scenario-based simulations to inform regional health planning and workforce policy in specialized care settings.

**Supplementary Information:**

The online version contains supplementary material available at 10.1186/s12942-026-00478-2.

## Introduction

In health geography, the spatial distribution and accessibility of healthcare facilities are widely recognized as fundamental determinants of regional healthcare provision. From emergency services to long-term care and outpatient treatment, spatial factors influence not only the utilization of services but also the equity in access across different population groups, as shown by a broad body of international research, including studies form Germany [1–5], Switzerland [6, 7], Italy [8], China [9] and England [10].

Although access to healthcare is a core principle of health policy, it remains unequally distributed in practice. Particularly in rural and structurally disadvantaged areas, disparities in infrastructure and service quality continue to result in unequal healthcare utilization. These spatial inequalities are associated with both acute and long-term health consequences for affected populations and present a challenge to the goal of providing needs-based, high-quality care across all regions. In response, several national policy frameworks have been implemented to restructure healthcare systems. In Germany, for example, the Hospital Structure Act [11], the Healthcare Strengthening Act [12] and the Act on the Reform of Emergency Care, which is currently under legislative review [13], all aim to improve quality and efficiency while ensuring that essential services remain accessible to local populations. A central challenge is to strike a balance between the benefits of centralization and the imperative of proximity-based care.

This balance is particularly relevant in the context of oncological care. On the one hand, specialized centers must meet structural and organizational requirements, including sufficient case numbers (which means a minimum catchment area) to ensure the viability of these facilities and thus guarantee quality-assured treatment. On the other hand, access to these services should not be hindered by excessive travel distances or other barriers.

For children and adolescents with cancer, treatment is typically concentrated in a small number of highly specialized units, often affiliated with large oncology centers at university hospitals or other maximal-care institutions. Due to the relatively low prevalence of cancer in this age group, such centralization is clinically necessary but often results in long travel distances and times for affected families. These journeys can pose a considerable burden for these families, as parents must coordinate not only the care of the ill child but also manage work responsibilities and, where applicable, the care for siblings at home. Comparable centralization–access tensions have also been documented in other forms of specialized pediatric care, particularly congenital heart disease [14, 15], neonatal inpatient care [16] and children with birth defects [17], where families may likewise face substantial travel burdens.

To better understand and plan for such spatially complex care structures, geographical modeling approaches have become increasingly important. While classical accessibility analysis focus primarily on GIS-supported travel time models [1–6], recent studies have emphasized the value of market area models that incorporate site-specific characteristics and empirically observed utilization behavior to estimate patient-to-hospital travel flows and delineating hospital catchment areas, respectively [18–23].

Building on this line of research, the present study develops and calibrates an extended Huff model [24] to analyze spatial utilization patterns in pediatric oncology care in Bavaria, Germany. Reflecting the principle of free choice of healthcare providers in Germany, the model is empirically parameterized based on real-world incident case numbers and integrates structural indicators, spatial proximity effects, and accessibility parameters to identify the key determinants of hospital choice. In doing so, it aims to provide a transferable and evidence-based tool to support regional health planning in specialized care contexts.

The paper is structured as follows. Section 2 explains the modeling concept and the study area. It describes the structural indicators incorporated into the model and presents the algorithm used to adapt the model to real-world data. Section 3 first presents and discusses the results of the model analysis. Subsequently, the developed model is applied to a simulation to examine how changes in structural indicators affect patient-to-hospital travel flows and the catchment areas of the hospitals under investigation. The conclusions and limitations of the study are then discussed (Sect. 4).

## Modeling approach

### Study area and patient data

**Fig. 1:**
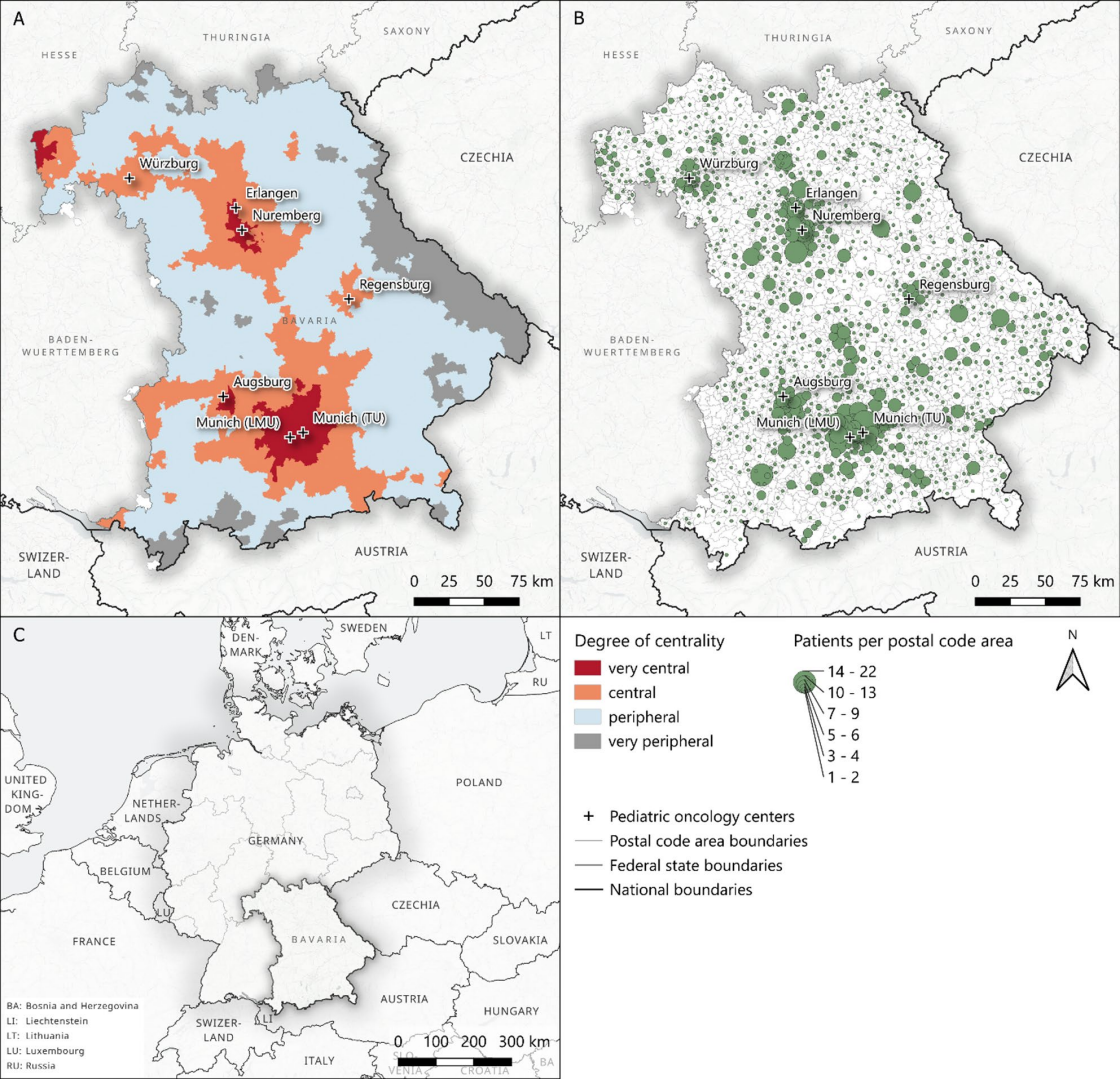
Study area and patient distribution. (**A**) Degree of centrality of pediatric oncology sites within Bavaria, Germany. (**B**) Distribution of pediatric oncology patients per postal code area, 2014–2023. (**C**) Location of Bavaria within Germany and Europe

The study area comprises the federal state of Bavaria, located in southeastern Germany (Fig. 1C). It covers approximately 70,500 km² and has a population of about 13 million inhabitants. Bavaria is characterized by a polycentric settlement structure that combines large urban centers such as Munich and Nuremberg with extensive rural regions of comparatively low population densities (Fig. 1A). The centrality categories shown in Fig. 1A are based on the official classification of the Federal Institute for Research on Building, Urban Affairs and Spatial Development (BBSR) [25].

In Bavaria, specialized pediatric oncology care is concentrated at seven hospital sites distributed across the state. These include university hospitals in Würzburg, Erlangen, Augsburg, Regensburg and Munich (two sites), as well as a dedicated pediatric facility in Nuremberg.

The empirical analysis is based on patient data from the German Childhood Cancer Registry covering the period from 2014 to 2023. Included were children and adolescents aged 0 to 17 years, who were residents of Bavaria at the time of cancer diagnosis and received treatment in one of the seven specialized centers. The places of residence of these patients are distributed across 1,264 of the 2,070 Bavarian postal code areas (Fig. 1B). In total, the dataset comprises 3,320 incident cases.

### Structural indicators

To account for structural differences between the seven hospital sites, we first extracted 16 potentially relevant indicators from official sources. These include the *Bavarian Hospital Plan* (BHP) [26], which serves as the regional regulatory framework for hospital infrastructure; the *Federal Hospital Atlas (Bundes-Klinik-Atlas*, BKA) [27], a nationwide online comparison portal; and the structured quality reports of each site (QR) [28–34], which are mandatory annual performance disclosures for all German hospitals. Additionally, we utilized data provided by the German Cancer Society (GCS) [35], the primary certification body for oncological centers in Germany. These indicators were subsequently assigned to five overarching structural dimensions (Table 1) and evaluated regarding their suitability for the analysis.

**Table 1:** Overview of structural indicators for the pediatric oncology hospital sites extracted from official data sources (BHP, BKS, QR, GCS)

Dimension	Indicators (Data Source)
Inpatient capacity	Inpatient beds (BHP)
	Inpatient beds (BKA)
	Inpatient beds (QR)
General treatment volumes	Inpatient cases (QR)
	Day-care cases (QR)
	Total cases (QR)
	Total cases (BKA)
Pediatric treatment volumes	Pediatric inpatient cases (QR)
	Pediatric day-care cases (QR)
	Pediatric total cases (QR)
	Pediatric total cases (BKA)
Staffing resources	Nursing staff ratio (BKA)
	Number of physicians (QR)
	Number of pediatric physicians (QR)
Oncology-specific accreditation	Number of cancer certificates (GCS)
	Oncology center accreditation (GCS)

Several indicators were excluded due to insufficient data quality or lack of conceptual fit. Variables related to treatment volumes were discarded because, in the authors’ view, they primarily reflect realized utilization (the outcome of the choice process) rather than a structural characteristic of the sites. In addition, treatment case numbers showed a strong correlation with bed capacity (Fig. S1), a proxy that has been firmly established in comparable structural analyses [18, 20, 21, 36, 37]. Accordingly, the number of inpatient beds was retained as the more consistent indicator for subsequent analyses.

In selecting the data source for this indicator, transparency and traceability were prioritized. The BHP was deemed unsuitable because multiple sites are aggregated into joint entries, preventing site-specific analysis. According to its documentation, the BKA data are based on hospital quality reports, yet they display minor inconsistencies with the reported figures and do not provide a clearly defined reference year. For this reason, figures were taken directly from the quality reports, as they represent the primary source, clearly indicate 2023 as reference year, and thus offer the most transparent basis for the analysis.

In addition to bed capacity, further indicators were incorporated. Two of these are the number of physicians and the number of pediatric physicians, defined as the number of full-time equivalent physicians employed at the hospital site or in the pediatric department, respectively.

The nursing staff ratio was also included. This measure reflects nursing workload relative to patient numbers, adjusted for case-specific care intensity. For analytical purposes, we used the reciprocal of the ratio to facilitate interpretation. For instance, a value of 40 indicates that one nurse was responsible for the equivalent of 40 average-care cases per year. Lower values therefore denote higher staffing levels and a more favorable care environment.

Oncology-specific accreditation was operationalized using two types of awards granted by the German Cancer Society: the number of organ-specific cancer certificates and the accreditation as an oncology center. The former documents compliance with defined quality standards in the treatment of individual tumor entities, whereas the latter requires the presence of several such certified organ units and attests to an overarching institutional structure that integrates multiple disciplines [38]. All used indicators are listed in Table 2.

**Table 2:** Structural indicators used in the analysis, including abbreviation, official site names and indicator values

Indicators			Inpatient Beds	Physicians	Pediatric Physicians	Nursing staff ratio	Cancer certificates	Oncology Center*
Abbreviation/Symbol			$\:{B}_{j}$	$\:{P}_{j}$	$\:PP$	$\:{NP}_{j}$	$\:{CC}_{j}$	$\:{OC}_{j}$
ID	Official German name (Site)	Site Location						
1	Universitätsklinikum Würzburg	Würzburg	1452	1056,7	62,3	41,2	12	1
2	Universitätsklinikum Erlangen	Erlangen	1462	883,0	76,8	50,7	14	1
3	Universitätsklinikum Augsburg (Medizincampus)	Augsburg	1531	720,4	47,2	50,7	8	1
4	Klinik Hallewiese – Cnopf’sche Kinderklinik	Nuremberg	285	160,2	16,4	39,6	0	0
5	Universitätsklinikum Regensburg	Regensburg	845	652,1	19,8	41,8	10	1
6	TUM Klinikum Rechts der Isar (Stammgelände)	Munich	1094	859,1	12,7	68,5	10	1
7	LMU Klinikum (Campus Großhadern)	Munich	1129	894,8	22,5	57,3	13	1

* Since Oncology Center is a dummy variable, it takes only binary values (1 = oncology center present, 0 = not present)

In addition to these site-specific structural indicators, spatial accessibility was considered as a separate dimension. To ensure a realistic representation of accessibility, it was defined based on car travel times rather than straight-line distance. This approach accounts for actual road network conditions, including speed limits and natural barriers.

Using the OpenRouteService API [39], travel times were computed between the centroids of all Bavarian postal code areas and the seven pediatric oncology hospital sites. The routing calculations are based on the OpenStreetMap (OSM) road network. These calculations primarily include roads permitted for cars, with priority given to motorways and trunk roads, while also accounting for one-way streets [40]. This routing service was selected because it provides flexible and up-to-date routing data and is entirely open source. Furthermore, previous comparative studies have shown that OSM coverage is comparable to that of commercial WebGIS and routing services and, in some cases, even surpasses it [41, 42].

### Model conceptualization

Similar to previous studies in spatial healthcare research, we employ a market area model for the delineation and segmentation of hospital catchment areas, specifically the Huff model [24, 43, 44]. Originally developed to estimate spatial customer flows to shopping centers, this model has since been adapted for the delineation and segmentation of catchment areas of healthcare facilities such as medical practices and hospitals [18–22, 36, 37, 45–47].

The original model proposed by Huff [24] aims to represent the utility of a retail location for the consumer, considering two explanatory variables: the size of the available locations and their accessibility to the customer origins. The utility of retail location $\:j$ for consumers in subarea $\:i$, $\:{U}_{ij}$, is defined as the product of the weighted variables, i.e., it is a multiplicative utility function:

1 $$\:{U}_{ij}={A}_{j}^{\gamma\:}{t}_{ij}^{-\lambda\:}$$

where $\:{A}_{j}$ is the size of location $\:j$, $\:{t}_{ij}$ is the travel time between customer origin $\:i$ and location $\:j$, and $\:\gamma\:$ and $\:\lambda\:$ are weighting parameters.

Huff [24] assumed that the size of a retail location has a positive effect, as a larger size increases the probability of finding the desired goods. However, it was assumed that this influence is sublinear $\:(0<\gamma\:<1)$, indicating diminishing marginal utility. Accessibility for consumers is represented by the travel time between $\:i$ and $\:j$. Here, Huff [24] postulated a superlinear negative effect $\:(\left|\lambda\:\right|>1)$, as travel time is interpreted as opportunity cost. The target variable of the model is the probability that customers from origin $\:i$ will visit the supply location $\:j$, denoted as $\:{p}_{ij}$. This probability is calculated as the ratio of the utility $\:{U}_{ij}$ to the sum of the utilities of all $\:J$ retail locations:

2 $$\:{p}_{ij}=\frac{{U}_{ij}}{{\sum\:}_{j=1}^{J}{U}_{ij}}$$

This choice probability is interpreted as the market share of location $\:j$ in origin $\:i$. By multiplying this market share with the local demand potential in region $\:i$, $\:{C}_{i}$, customer flows to retail location $\:j$ from region $\:i$, $\:{E}_{ij}$, can be estimated:

3 $$\:{E}_{ij}={p}_{ij}{C}_{i}$$

The total market area of supply location $\:j$, $\:{T}_{j}$, is the sum of all customer flows from $\:I$ origins [44]:

4 $$\:{T}_{j}=\sum\:_{i=1}^{I}{E}_{ij}$$

In healthcare research, this model has been adapted to locations of healthcare facilities, particularly because the aspect of accessibility or travel time holds similarly high (or even higher) importance. Which “attractiveness” variable $\:{A}_{j}$ is chosen depends on the type of healthcare location being studied. Typically, a proxy for the capacity of a healthcare location is used. When analyzing the supply of physician practices, either a physician practice is counted as a constant value of 1 or the number of physician practices per region is used [19, 22, 45–47]. When analyzing hospital catchment areas, the number of beds (total or for a specific medical specialty) is typically used as the “attractiveness” value [18, 20, 21, 36, 37]. Similar to the original Huff model, travel times are typically used. In most cases, these analyses primarily aim to create the most realistic representation of accessibility possible. In this case, travel time $\:{t}_{ij}$ is sometimes weighted with an exponential function [19, 20] or a logistic function [18, 22, 46, 47] rather than a power function, as in the original model.

However, we are considering a specific case of medical care (pediatric oncology) and also want to build a model that approximately explains patient choice and the corresponding hospital catchment areas. Therefore, we need to make substantial extensions to the approaches mentioned above. We aim to capture the quality of a hospital in a multidimensional manner by expanding the number of explanatory variables. For this reason, we tested combinations of several of the indicators described above as explanatory variables (see section “Fitting procedure”). However, in each combination, we included bed capacity as a basic capacity indicator, analogous to previous literature on market area analyses of hospitals [18, 20, 21, 36, 37].

Furthermore, we have to take into account the spatial structure of the Bavarian hospitals, especially because certain hospitals (notably Erlangen and Nuremberg as well as the two sites in Munich) are located very close to each other (see Fig. 1). Therefore, following the *Competing Destinations Model* by Fotheringham [48] which is an extension of the Huff model, we include the “relative location” of a hospital $\:j$ to the other hospitals. This additional variable, $\:{C}_{j}$, is thus a measure of the spatial concentration (clustering) of the individual hospital relative to all other hospitals, called “clustering variable” hereafter. Analogous to Fotheringham [48], we define $\:{C}_{j}$ as follows:

5 $$\:{C}_{j}=\sum\:_{k=1,\:\:\:k\ne\:j}^{J-1}{B}_{k}^{\psi\:}{t}_{jk}^{-\varphi\:}$$

where $\:J-1$ denotes the number of hospitals except hospital $\:j$, $\:{B}_{k}$ and $\:{t}_{jk}$ are the bed capacity of hospital $\:k$ and the travel time between hospital $\:j$ and $\:k$, respectively, and $\:\psi\:$ and $\:\varphi\:$ are the corresponding weighting coefficients. For the calculation of $\:{C}_{j}$, two weighting parameters are required. As these parameters are unknown and no reference values are available from previous studies, we set them to $\:\psi\:=1$ and $\:\varphi\:=2$, corresponding to commonly used default weighting exponents in market area models [see, e.g. 49, 50]. This implies a linear size effect and a relatively strong distance decay effect [49].

Since the combination of partial utilities was not determined in advance, our generalized utility function is thus:

6 $$\:{U}_{ij}={B}_{j}^{\alpha\:}\prod\:_{m=1}^{M}{g}_{m}({X}_{jm};{{\Theta\:}}_{m})*f\left({t}_{ij}\right)$$

where $\:{B}_{j}$ is the bed capacity of hospital $\:j$, and $\:\alpha\:$ is the corresponding weighting coefficient. The term $\:{g}_{m}(\cdot\:)$ denotes the transformation function applied to the explanatory variable $\:{X}_{jm}$ of hospital $\:j$. The corresponding parameter $\:{{\Theta\:}}_{m}$ captures the strength of influence that this attribute exerts on the attractiveness of hospital $\:j$. Depending on the type of the variable, $\:{{\Theta\:}}_{m}$ is either applied as an exponent in a power function $\:\left({x}^{{{\Theta\:}}_{m}}\right)$ or as a slope parameter in an exponential function $\:\left(\mathrm{exp}\left({{\Theta\:}}_{m}x\right)\right)$. As we have five structural variables and one cluster variable, the index $\:m$ ranges from 1 to 6. The type of travel time weighting, $\:f\left({t}_{ij}\right)$, is described below.

Following earlier studies on Huff model analysis of hospital catchment areas [18, 21, 36, 37], we retain a power function as the weighting function for bed capacity $\:{B}_{j}$, number of physicians $\:{P}_{j}$, number of pediatric physicians $\:{PP}_{j}$, and nursing staff ratio $\:{N}_{j}$. However, this is not applicable for the number of cancer certificates $\:{CC}_{j}$ and oncology center accreditation $\:{OC}_{j}$, as both variables may take values equal to zero, especially because $\:{OC}_{j}$ is a dummy variable. Thus, these variables are weighted with an exponential function. The clustering indicator $\:{C}_{j}$ is weighted with a power function, according to the model proposal and related applications [48, 50]. Following previous studies on the accessibility of healthcare locations [18, 22, 46, 47], we use a logistic function for the weighting of travel time $\:{t}_{ij}$.

7 $$\:f\left({t}_{ij}\right)=\frac{1}{1+\mathrm{e}\mathrm{x}\mathrm{p}\left({\lambda\:}_{1}\left({t}_{ij}-{\lambda\:}_{2}\right)\right)}$$

Here, $\:{\lambda\:}_{1}$ denotes the slope parameter, determining how sharply accessibility declines with increasing travel time, while $\:{\lambda\:}_{2}$ specifies the inflection point of the function, that is, the travel time at which accessibility is reduced to 50% of its maximum value.

### Fitting procedure

The weighting parameters in the Huff model have a very strong influence on the estimated interaction probabilities because the model is nonlinear in its parameters. Therefore, empirically estimating these parameters using real data has been a research area for decades. Huff [24] already proposed an algorithm for the empirical determination of the distance decay parameter *λ*. A transformation of the model into an econometric model by Nakanishi and Cooper [51] allows this to be done relatively conveniently, but requires empirically determined interactions or market shares [49, 52]. In our case, however, no data on empirical hospital catchment areas are available, i.e., it is unknown which hospitals are visited from which postal code areas. Therefore, an empirical determination of the weighting parameters is only possible based on the total number of patients available. Assuming that $\:{T}_{j}$ is known, several approaches have been developed for parameterizing or optimizing the Huff model. These include iterative adjustments of the attractiveness indicator [49, 53] and parameter estimations using different methods of nonlinear regression and nonlinear optimization, respectively [50, 54, 55].

In our case, following Orpana and Lampinen [50] and others, we opted for a maximum likelihood estimation. Here, the negative log-likelihood is minimized, which is calculated as the sum of logged squared residuals for $\:J$ hospitals:

8 $$\:LL=-\sum\:_{j=1}^{J}\mathrm{l}\mathrm{o}\mathrm{g}{\left(\right({T}_{j,\:\:obs}-{T}_{j,emp})}^{2})$$

where $\:{T}_{j,obs}$ equals the observed total number of patients of hospital $\:j$ and $\:{T}_{j,emp}$ is the expected total number of patients which is calculated using the Huff model.

Because in our case the combination of partial utilities was not determined a priori, we estimated a series of alternative model specifications with different sets of explanatory variables (see Table 2 and Formula 6). For each specification, a grid search with three starting values per parameter was performed (Table 3). In each iteration, interaction probabilities and total market areas were calculated according to Formulas (2)–(4), combined with the utility function in Formula (6). For each iteration, goodness-of-fit metrics were calculated by comparing observed and predicted values. These metrics were computed automatically using the software package employed in this study (see below) and included R-squared, mean absolute error (MAE), and mean absolute percentage error (MAPE). Following the approaches in previous studies on Huff model optimization [20, 49, 54], we used the MAPE as the primary criterion for selecting the best-performing model:

9 $$\:MAPE=\frac{1}{J}\sum\:_{j=1}^{J}\left|\frac{{T}_{j,obs}-{T}_{j,emp}}{{T}_{j,obs}}\right|*100$$

This goodness-of-fit metric represents the average percentage deviation of the model’s prediction from the observed values, regardless of whether the deviation is positive or negative. Additionally, local absolute percentage errors (APE) were calculated to determine the share of hospitals predicted within different deviation thresholds (5%, 10%, 15%, 20%, etc.):

10 $$\:APE=\left|\frac{{T}_{j,obs}-{T}_{j,emp}}{{T}_{j,obs}}\right|*100$$

We have chosen an optimization algorithm which requires starting values and allows for parameter bounds, thereby ensuring that the empirically estimated parameters always lie within a predefined range. Both inputs may influence the outcome, making it necessary to use plausible starting values and bounds. We formulated these based on previous studies and theoretical considerations. Previous studies have found an empirical weighting exponent for bed capacity greater than 0.5, but mostly less than 1, which corresponds to a sublinear positive impact on patient-to-hospital travel flows [18, 20, 21]. We therefore limited the parameter to values between 0.1 and 0.9 and set values between 0.3 and 0.7 as starting values.

The nursing staff ratio reflects the relationship between the number of patients and available nursing personnel, thus providing an indication of staffing levels within a facility. Higher values may point to limited personnel resources, but may also result from high case numbers and, consequently, high hospital utilization. We interpret higher nursing staff ratios as a negative attribute of a hospital, regardless of whether they stem from lower staffing levels, higher patient volumes, or both. This assumption is supported, among other things, by findings in the health service research showing that high occupancy rates or high nurse-to-patient ratios are associated with poorer patient outcomes [56, 57]. Consequently, all starting values for the weighting coefficients of the nursing staff ratio were set in the negative range, specifically between − 0.7 and − 0.3, with parameter bounds of −0.9 and − 0.1. Since the number of cancer certificates varies only slightly between hospitals, and all but one of the hospitals in the study area have an oncology center, we assumed that the influence would be relatively small. The starting values and bounds for these two exponentially weighted variables were therefore set relatively low.

Regarding the clustering variable, we assumed that proximity to another hospital has a negative effect, meaning that competitive effects predominate. This is to be expected because visits to multiple hospitals are rarely linked, but rather the decision for one hospital excludes the others. Similar results have also been found with regard to service locations [50]. Table 3 provides an overview of the start values and bounds used for the grid search within the fitting iterations.

**Table 3:** Overview of starting values and parameter bounds used in the grid search procedure

Variable	Parameter	Start values			Bounds	
Bed capacity	$\:\alpha\:$	0.3	0.5	0.7	0.1	0.9
Nursing staff ratio	$\:{\theta\:}_{1}$	−0.7	−0.5	−0.3	−0.9	−0.1
Physicians	$\:{\theta\:}_{2}$	0.3	0.5	0.7	0.1	0.9
Pediatric physicians	$\:{\theta\:}_{3}$	0.3	0.5	0.7	0.1	0.9
Cancer certificates	$\:{\theta\:}_{4}$	0.1	0.3	0.5	0.6	0.01
Oncology center	$\:{\theta\:}_{5}$	0.1	0.3	0.5	0.6	0.01
Clustering	$\:{\theta\:}_{6}$	−0.5	−0.25	0.0	−0.75	0.25
Travel time	$\:{\lambda\:}_{1}$	−0.5	−0.25	−0.1	−0.7	−0.01
	$\:{\lambda\:}_{2}$	20	25	30	10	40

All Huff model analyses, including fitting, were performed in Python v.3.12.11 [58] using the *huff* package v.1.5.14 [59]. Model parameters were estimated by minimizing the negative log-likelihood presented in Formula (8) using the *trust-constr* (Trust-Region Constrained). This minimization algorithm was developed by Byrd et al. [60] and implemented in the Python package *scipy.optimize* [61].

## Results and discussion

### Baseline model and goodness of fit

As outlined above, we estimated multiple model specifications with different sets of explanatory variables and compared their performance using the MAPE. The specification with the lowest MAPE was selected as our baseline model.

This model included four “attractiveness” variables (bed capacity, nursing staff ratio, cancer certificates, and oncology cancer accreditation), one clustering variable, and a logistic function for travel time. The corresponding utility function is defined as:

11 $$\:{U}_{ij}={B}_{j}^{\alpha\:}{N}_{j}^{\beta\:}\mathrm{exp}\left(\gamma\:CC\right)\mathrm{e}\mathrm{x}\mathrm{p}\left(\delta\:OC\right){C}_{j}^{\eta\:}{\left(1+\mathrm{exp}\left({\lambda\:}_{1}\left({t}_{ij}-{\lambda\:}_{2}\right)\right)\right)}^{-1}$$

The baseline model achieved a MAPE of 5.85% and a $\:{R}^{2}$ of 0.89. Total patient numbers were reproduced with deviations below $\:\pm\:$15%, and for five of the seven hospitals (71.43%) deviations were even below $\:\pm\:$10%. Table 4 presents the goodness-of-fit statistics for the baseline model and two alternative specifications with comparatively good performance.

Direct benchmarks for interpreting our MAPE are scarce. However, Latruwe et al. [20], who also modeled hospital catchment areas and compared predicted and observed data, report MAPEs ranging from 9.59% to 17.76% across four study areas. By comparison, the MAPE of 5.85 achieved here indicates a relatively strong model fit.

**Table 4:** Goodness-of-fit metrics for three parametrized extended Huff models

Model	Baseline model	Alternative model 1	Alternative model 2
Variables in utility function	Bed capacity $\:\left({B}_{j}\right)$ Travel time $\:\left({t}_{ij}\right)$ Nursing staff ratio $\:\left({N}_{j}\right)$ Cancer certificates $\:\left({CC}_{j}\right)$ Oncology center $\:\left({OC}_{j}\right)$ Clustering $\:\left({C}_{j}\right)$	Bed capacity $\:\left({B}_{j}\right)$ Travel time $\:\left({t}_{ij}\right)$ Nursing staff ratio $\:\left({N}_{j}\right)$ Cancer certificates $\:\left({CC}_{j}\right)$ Pediatric physicians $\:\left({PP}_{j}\right)$ Clustering $\:\left({C}_{j}\right)$	Bed capacity $\:\left({B}_{j}\right)$ Travel time $\:\left({t}_{ij}\right)$ Nursing staff ratio $\:\left({N}_{j}\right)$ Cancer certificates $\:\left({CC}_{j}\right)$ Pediatric physicians $\:\left({PP}_{j}\right)$
$\:{R}^{2}$	0.89	0.86	0.83
$\:MSE$	18.68	24.16	28.78
$\:RMSE$	4.32	4.92	5.37
$\:MAE$	3.20	3.84	4.01
$\:\boldsymbol{M}\boldsymbol{A}\boldsymbol{P}\boldsymbol{E}$	**5.85**	**7.20**	**7.60**
$$\:APE$$			
$\:<5\%$	42.86	57.14	42.86
$\:<10\%$	71.43	57.14	71.43
$\:<15\%$	100.00	100.00	85.71
$\:<20\%$	100.00	100.00	100.00

### Parameter estimates

In our baseline model, seven parameters were estimated: five associated with explanatory variables and two with the travel-time function. With three starting values per parameter, the grid search involved $\:{3}^{7}=\mathrm{2,187}$ iterations. Table 5 reports the estimated coefficients. Furthermore, it is important to note that the parameter estimates are not directly comparable across variables, as different functional forms – namely power, exponential, and logistic transformations – were applied depending on the nature of the explanatory variables. Nonetheless, individual coefficients can be compared to analogous estimates reported in previous studies using the same type of transformation function.

**Table 5:** Estimated coefficients of our baseline model

Variable in utility function	Weighting parameter	Parameter value
Bed capacity $\:\left({B}_{j}\right)$	$\:\alpha\:$	0.404
Nursing stuff ratio $\:\left({N}_{j}\right)$	$\:\beta\:$	−0.619
Cancer certificates $\:\left({CC}_{j}\right)$	$\:\gamma\:$	0.042
Oncology center $\:\left({OC}_{j}\right)$	$\:\delta\:$	0.305
Clustering $\:\left({C}_{j}\right)$	$\:\eta\:$	−0.049
Travel time $\:\left({t}_{ij}\right)$	$\:{\lambda\:}_{1}$	20.006
	$\:{\lambda\:}_{2}$	−0.078

As expected, all hospital indicators except for the staffing ratio, display positive coefficients, indicating that an increase in, for example, bed capacity raises the likelihood of patients choosing this hospital. For the staffing ratio, it should be noted that the inverse of it is included in the utility function, meaning that its negative coefficient implies that better staffing levels increase the probability of interaction.

For most coefficients, no direct reference values exist, as previous studies have rarely combined multiple utility indicators for hospitals and none have focused on pediatric oncology anyway (see section "Structural indicators"). However, the coefficient for bed capacity $\:\left(\alpha\:\right)$ can be compared to some previous work. Jia et al. [21] interpret $\:\alpha\:$ as the elasticity of hospital capacity and assume, similar to the original Huff model, a sublinear impact. They find the best model with a parameter of $\:\alpha\:=0.82$. Latruwe et al. [20] differentiate size coefficients by patient age groups and find values of $\:\alpha\:$ between 0.78 and 1.22. Bai et al. [18] report a parameter of 0.88 for obstetric hospitals. Our value of $\:\alpha\:=0.404$ is therefore lower, but also reflects a sublinear effect of capacity.

As expected, the coefficient for the clustering variable $\:\left(\eta\:\right)$ is negative, indicating that spatial proximity to another hospital with a pediatric oncology unit reduces the probability of interaction. From an economic perspective, this can be interpreted as a spatial competition effect, analogous to previous studies with market area models regarding retail and service locations [50, 55]. Following these studies, a positive value of $\:\eta\:$ would indicate agglomeration benefits, which may be plausible for retail businesses but not for hospitals, where visits are not typically combined. To our knowledge, this effect has not yet been investigated for healthcare facilities.

The results for the logistic distance-decay function cannot be directly compared with other studies. However, Latruwe et al. [20], for instance, also found best model fits using a logistic function. We therefore assume that this functional form best captures the decelerating effect of travel time.

### Estimated catchment areas

**Fig. 2:**
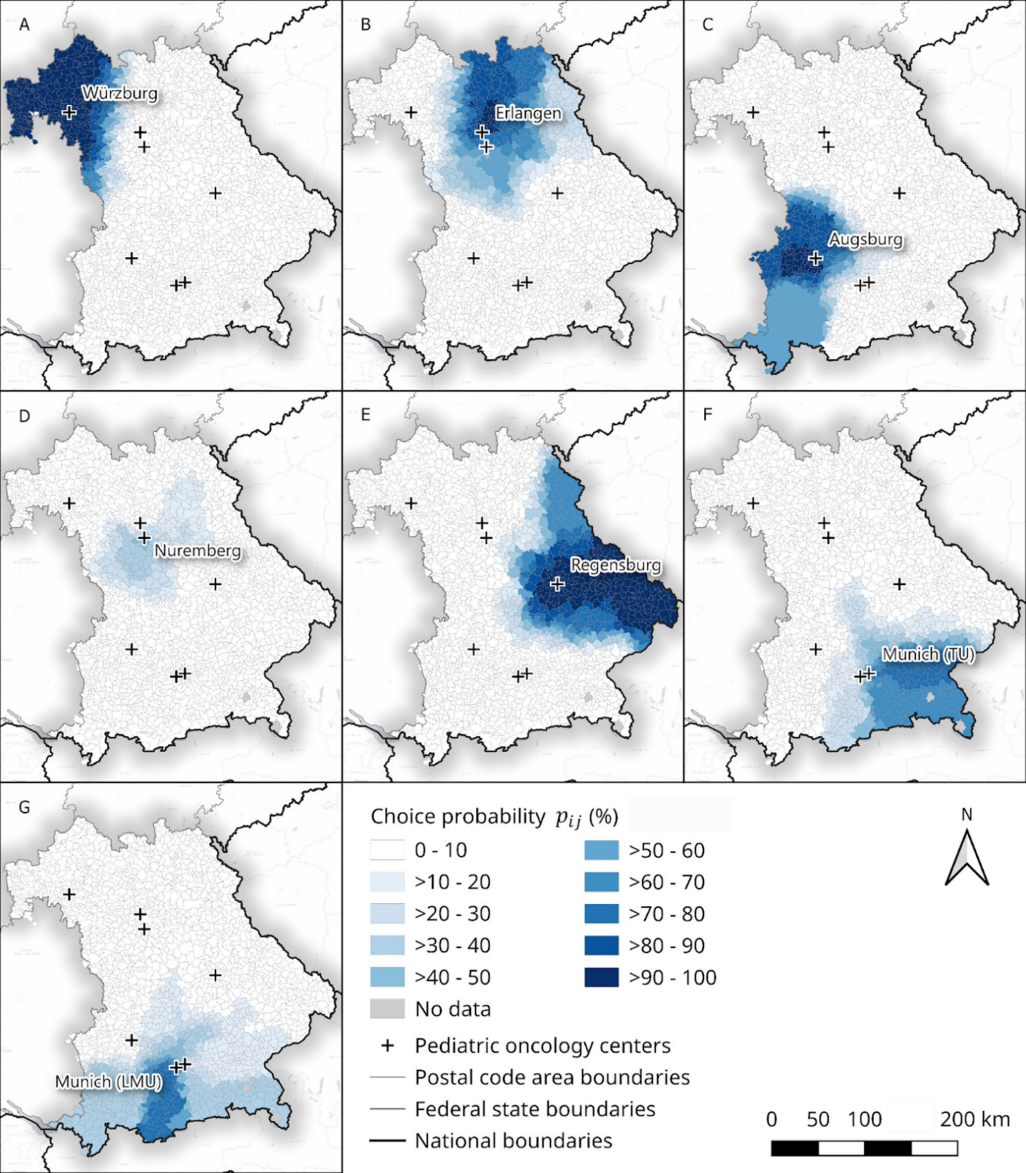
Modeled choice probabilities ($\:{p}_{ij}$) for the seven pediatric oncology hospitals in Bavaria. (**A**) Würzburg, (**B**) Erlangen, (**C**) Augsburg, (**D**) Nuremberg, (**E**) Regensburg, (F) Munich (TU) and (**G**) Munich (LMU)

Based on the empirically parameterized model, we calculated interaction probabilities $\:{p}_{ij}$ for the seven hospitals across all Bavarian postal code areas (Formula (2) using the utility function from Formula (11)). The results are presented in Fig. 2, which illustrates spatially distinct yet partly overlapping catchment areas.

As expected, probabilities decline with increasing distance from each hospital site. Around Würzburg, Regensburg, and Augsburg, compact and largely contiguous high-probability zones emerge with only limited overlap with adjacent areas. South of Augsburg, however, a marked overlap with the catchment area of the LMU Munich becomes evident. Erlangen forms a coherent northern catchment area but overlaps with Nuremberg to the south, while Nuremberg itself shows only limited spatial reach and comparatively weak probabilities. The Munich metropolitan area shows the strongest interactions. The catchment areas of LMU and TU intersect substantially, with LMU extending predominantly towards the southwest and TU towards the southeast, reflecting a high degree of overlap.

**Fig. 3:**
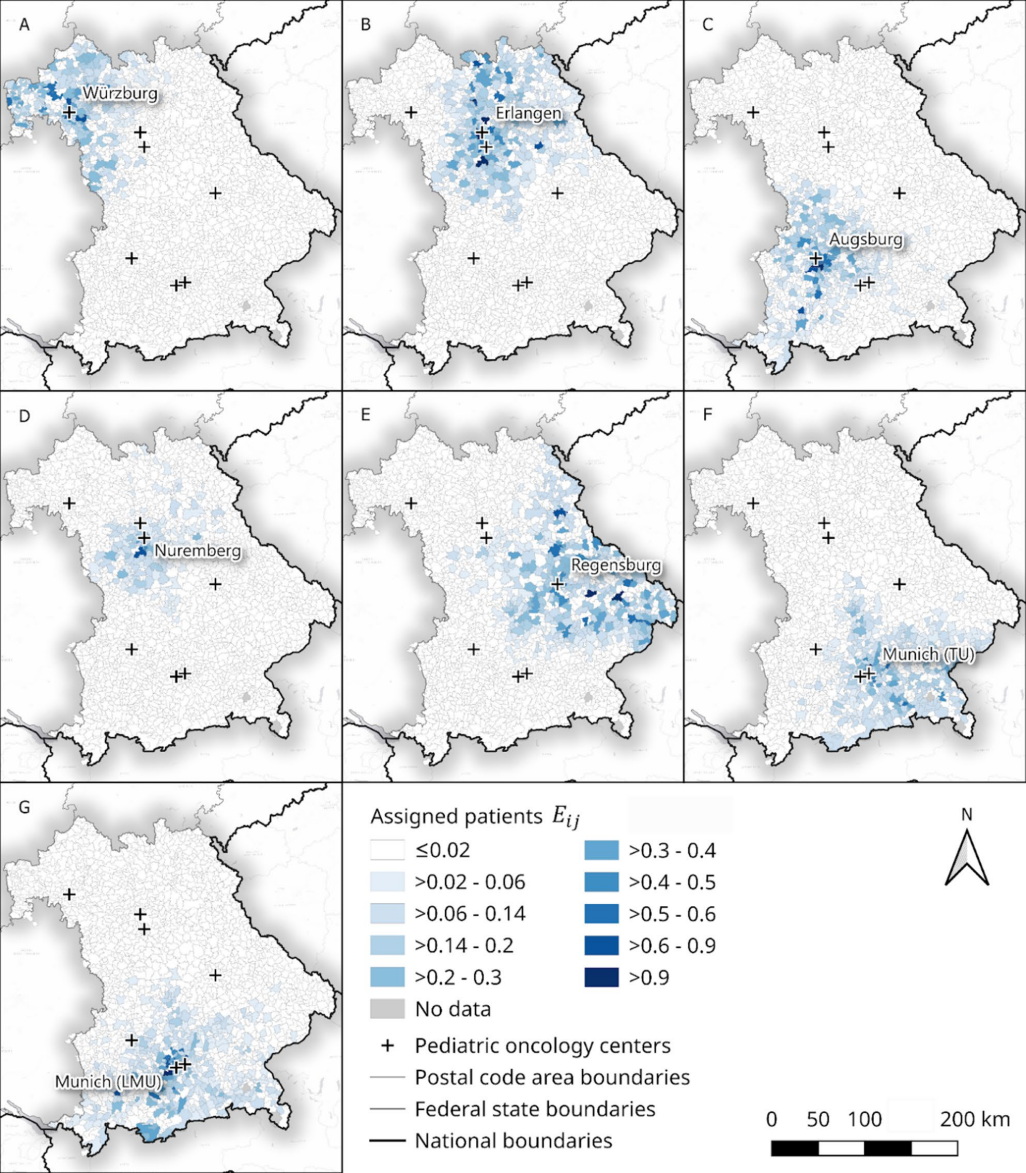
Modeled patient-to-hospital travel flows ($\:{E}_{ij}$) assigned to the seven pediatric oncology hospitals in Bavaria. (**A**) Würzburg, (**B**) Erlangen, (**C**) Augsburg, (**D**) Nuremberg, (**E**) Regensburg, (**F**) Munich (TU) and (**G**) Munich (LMU)

Figure 3 further illustrates the resulting patient-to-hospital travel flows $\:{E}_{ij}$ (Formula (3)) which appear more dispersed and fragmented than the modeled probabilities, underlining the influence of overlapping catchment areas.

### Scenario analysis

With the baseline model established, we now can examine how changes in hospital-related factors would affect spatial interaction patterns. This enables us to simulate a variety of hypothetical scenarios, such as an increase in bed capacity at a given site, chances in nursing staff ratio, or, in more extreme cases, the closure of an entire hospital.

As an application example, we here focus on the development of nursing staff ratios at the two Munich sites. It is assumed that staffing levels relative to patient numbers will decrease further, thereby deliberately reducing the relative “attractiveness” of the Munich hospitals. Already today, both sites display the lowest nursing staff ratios compared to the other hospitals (see Table 2). Current studies on workforce development project a further aggravation of the situation in the coming years, driven by increasing service demand, declining staff numbers due to rising retirement rates, and decreasing training capacities [62]. This challenge is amplified by the exceptionally high costs of living in Munich, which structurally hampers the recruitment and retention of nursing staff despite high demand. For example, rental prices in Munich are about 51% above the national average [63]. In combination with salaries that are less competitive in relation to local living costs and with high workloads, this fosters outward migration tendencies [64], which can be represented in the model as a spatially uneven reduction of the nursing staff ratio. In our scenario, we assume a 20% reduction of the ratio at the two Munich sites, while ratios at the remaining sites remain unchanged.

**Fig. 4:**
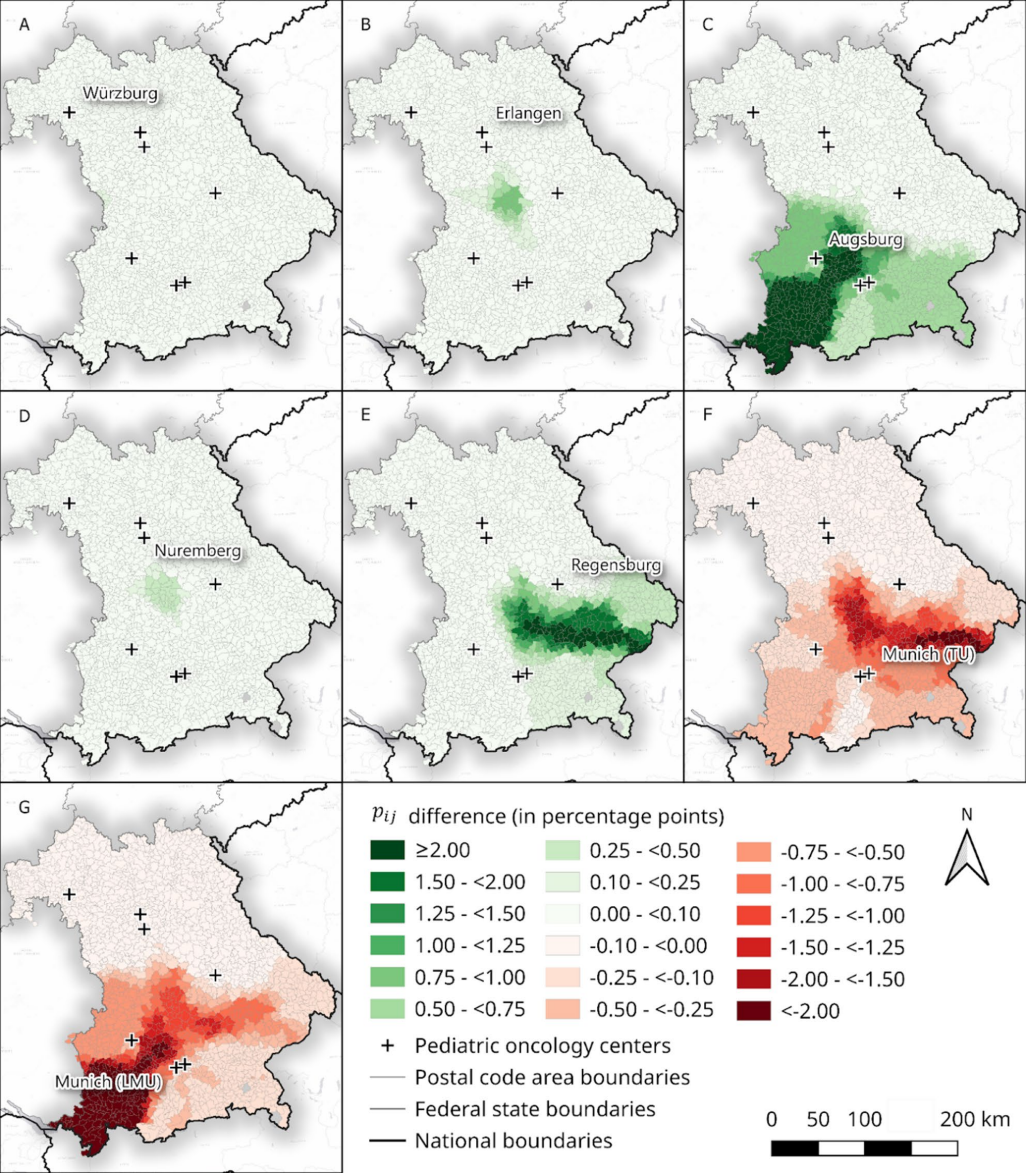
Differences in modeled choice probabilities $\:({p}_{ij}$) between the baseline model and the Munich staffing scenario, expressed in percentage points. Green areas indicate probability gains for the respective hospital, while red areas indicate losses. (**A**) Würzburg, (**B**) Erlangen, (**C**) Augsburg, (**D**) Nuremberg, (**E**) Regensburg, (**F**) Munich (TU) and (**G**) Munich (LMU)

The results of the scenario are summarized in Table 6 and illustrated in Fig. 4. Compared to the baseline, the assumed 20% reduction of the nursing staff ratio at the two Munich sites reduces their modeled “attractiveness” and redistributed patient-to-hospital travel flows to other hospitals.

Both Munich sites show declining totals, with − 2.0% points (pp) for TU and − 2.5 pp for LMU. These reductions are offset primarily by gains at Augsburg (+ 3.5 pp) and Regensburg (+ 1.3 pp), while Würzburg, Erlangen and Nuremberg remain largely unaffected (≤ 0.2 pp). The spatial redistribution is also evident in Fig. 4. Losses for the Munich sites are relatively modest in areas where they already exhibited strong dominance (high $\:{p}_{ij}$), but become more pronounced in zones where catchments previously overlapped with those of Augsburg or Regensburg (see Fig. 2). In these transitional areas, where patient-to-hospital travel flows were already shared between sites, a larger share now shifts towards the neighboring hospitals.

**Table 6:** Differences in modeled patient totals (∆Tj) between the baseline model and the Munich staffing scenario

$$\:(\varDelta\:{T}_{j})$$	
Site location	$\:\varDelta\:{T}_{j}$ (in percentage points)
Würzburg	0.0
Erlangen	0.2
Augsburg	3.5
Nuremberg	0.2
Regensburg	1.3
Munich (TU)	−2.0
Munich (LMU)	−2.5

This pattern suggests that changes in staffing conditions at given sites are reflected more strongly on peripheral parts of their catchments than in core zones. Areas with already high interaction probabilities remain relatively stable, whereas overlapping and transitional areas appear more responsive to variations in relative “attractiveness”. It should be noted that the Munich locations are already relatively poorly equipped with nursing staff, which is why the effect predicted by the model is comparatively small.

## Conclusions and limitations

In our study, we developed and empirically calibrated an extended Huff model to analyze spatial utilization patterns in specialized healthcare. Using the case of pediatric oncology care in Bavaria, we demonstrated that incorporating structural site characteristics, a spatial clustering variable, and a realistic distance-decay function significantly improves model performance (MAPE: 5.85%). The resulting baseline model not only enables the mapping of hospital catchment areas and choice probabilities but can also be used directly for planning purposes. In an illustrative scenario analysis, we further showed how structural changes, such as a reduction in staffing levels at selected sites, can influence the distribution of patient-to-hospital travel flows.

Like any model-based analysis, our approach is subject to several limitations. The incident case numbers are based exclusively on patients residing in Bavaria who were treated at one of the seven investigated hospitals, so cross-border treatment flows are not captured. While this restriction narrows the empirical scope, it also ensures a consistent and internally closed system (equilibrium) that provides a robust basis for analysis. As an equilibrium model, it assumes that the total demand (patients) is fully integrated and distributed across the available supply locations (hospitals) [49]. In practice, this assumption is challenged by a lack of data regarding cross-border patient flows, specifically, Bavarian residents treated outside the state and non-residents treated within it. Despite these boundary effects, the current dataset remains the only viable basis for analysis. Methodologically, the occurrence of zero empirical cases $\:({C}_{i}=0)$ in certain postal code areas does not signify data gaps; rather, it is a direct consequence of the low incidence of pediatric cancer, the ten-year observation period, and the high spatial resolution.

Furthermore, the spatial analysis should be critically examined regarding the Modifiable Areal Unit Problem (MAUP). While the MAUP was comprehensively described early on as a fundamental statistical problem of spatial aggregation [65], newer studies emphasize its specific implications for geospatial health research [66]. Accordingly, the confinement of population data to administrative boundaries introduces an inherent uncertainty, as the precise distribution of the population-at-risk within these units remains hidden. As a result, findings might vary if alternative levels of aggregation, such as grid-based data or municipal boundaries, had been employed.

Another limitation arises from the calculation of travel times using geometric centroids. Although this approach is methodologically common [4, 20, 55, 67], it may lead to distortions in unevenly populated areas, for instance when centroids fall in unrepresentative locations such as forests. Moreover, travel times were modeled under the assumption of optimal traffic conditions, which likely underestimate real-world variability caused by congestion, for instance. A much better approach would be to use small-scale grids for which population data are available [6]. However, patient data are not available at this spatial aggregation level, so it would not have been possible for us to use this scale level.

A temporal effect could also potentially have introduced bias. While the patient data cover the period from 2014 to 2023, the structural indicators of the hospitals are exclusively based on data from 2023. Consequently, potential temporal variations in structural characteristics, such as staffing levels, are not fully captured in the model. However, longitudinal data that would allow for a temporally consistent analysis are not available for two primary reasons. First, the *Federal Hospital Atlas* was only launched in 2024. Second, according to the official reporting body, hospital quality reports for the years 2019 to 2022 are incomplete and therefore of limited suitability for robust analyses [68]. However, we assume that these structural characteristics remained relatively stable over the observation period and are therefore unlikely to substantially affect the results.

Finally, the modeled catchment areas in Munich (LMU/TU) are only partially realistic: Instead of a clear spatial division into southeastern and southwestern zones, a stronger degree of overlap is more likely. This highlights the need for further refinement of the model, as spatial proximity and institutional characteristics appear to shape actual care patterns more strongly than the current specification can capture.

Despite these limitations, our model provides a robust and adaptable framework for analyzing spatial utilization patterns in specialized healthcare. A key strength of our model lies in its flexibility and transferability. By allowing for the integration of context-specific indicators and functional forms, the approach can be adapted to other medical specialties, healthcare systems, or regional planning contexts. This makes it particularly valuable in settings where empirical data on patient-to-hospital travel flows or catchment areas are limited.

In addition, the model allows for scenario-based simulations by systematically varying selected indicators. This enables the assessment of how structural adjustments such as changes in staffing levels or bed capacities may affect patient distribution and interaction patterns. Accordingly, our model offers a practical and transferable tool for planners and policymakers to anticipate spatial effects of system changes and support evidence-based decision-making in specialized care.

## Electronic Supplementary Material

Supplementary material 1

## Data Availability

All structural indicators for the hospital sites analyzed in this study are publicly available and can be obtained from the sources cited in references [26–35]. The patient data required for the analysis (pediatric oncological incident case numbers per postal code area) were provided by the German Childhood Cancer Registry (DKKR). These data are not publicly available, and the authors are not authorized to share them with third parties. However, in justified cases, the data can be requested directly from the DKKR (www.kinderkrebsregister.de).

## Abbreviations

APE: Absolute Percentage Error
BKA: Bundes-Klinik-Atlas (Federal Hospital Atlas)
GCS: German Cancer Society
MAE: Mean Absolute Error
MAPE: Mean Absolute Percentage Error
MSE: Mean Squared Error
MAUP: Modifiable Areal Unit Problem
OSM: OpenStreetMap
QR: Quality Report

## References

[1] Augustin J, Erasmi S, Reusch M, Augustin M. Methods of analyzing regional dermatological care as exemplified by the city of Hamburg. JDDG J Dtsch Dermatol Ges. 2015;13:661–71. 10.1111/ddg.12626. 26110724 10.1111/ddg.12626

[2] Neumeier S. Regional distribution of ambulant nursing services in Germany. A GIS accessibility analysis. Raumforsch Raumord. 2016;74:339–59. 10.1007/s13147-016-0409-4.

[3] Rauch S, Stangl S, Haas T, Rauh J, Heuschmann PU. Spatial inequalities in preventive breast cancer care: a comparison of different accessibility approaches for prevention facilities in Bavaria, Germany. J Transp Health. 2023;29:101567. 10.1016/j.jth.2023.101567.

[4] Stentzel U, Piegsa J, Fredrich D, Hoffmann W, van den Berg N. Accessibility of general practitioners and selected specialist physicians by car and by public transport in a rural region of Germany. BMC Health Serv Res. 2016;16:587. 10.1186/s12913-016-1839-y. 27756338 10.1186/s12913-016-1839-y PMC5070365

[5] Wieland T. Modellgestützte Verfahren und „big (spatial) data“ in der regionalen Versorgungsforschung I [Model-based methods and “big (spatial) data” in regional heath services research I]. Monit Versorg. 2018;11:41–5. 10.24945/MVF.02.18.1866-0533.2072.

[6] Rauch S, Rakic M, Hengartner H, Elger B, Rost M. Access to paediatric oncology centres in Switzerland: Disparities across rural–urban and Swiss-foreigners cohorts. Eur J Cancer Care (Engl). 2022;31. 10.1111/ecc.13679. 10.1111/ecc.13679 PMC9788087 35942909

[7] Haynes AG, Wertli MM, Aujesky D. Automated delineation of hospital service areas as a new tool for health care planning. Health Serv Res. 2020;55:469–75. 10.1111/1475-6773.13275. 32078171 10.1111/1475-6773.13275 PMC7240760

[8] Pecoraro F, Cellini M, Luzi D, Clemente F. Analysing the intra and interregional components of spatial accessibility gravity model to capture the level of equity in the distribution of hospital services in Italy: do they influence patient mobility? BMC Health Serv Res. 2024;24:973. 10.1186/s12913-024-11411-3. 39180078 10.1186/s12913-024-11411-3 PMC11342588

[9] Dong X, Wang Y. The geography of healthcare: mapping patient flow and medical resource allocation in China. Econ Hum Biol. 2024;55:101431. 10.1016/j.ehb.2024.101431. 39326297 10.1016/j.ehb.2024.101431

[10] Challen RJ, Griffith GJ, Lacasa L, Tsaneva-Atanasova K. Algorithmic hospital catchment area estimation using label propagation. BMC Health Serv Res. 2022;22:828. 10.1186/s12913-022-08127-7. 35761225 10.1186/s12913-022-08127-7 PMC9235278

[11] Bundesministerium der Justiz und für Verbraucherschutz [Federal Ministry of Justice and Consumer Protection]. Gesetz zur Reform der Strukturen der Krankenhausversorgung (Krankenhausstrukturgesetz – KHSG) [Act for the Reform of Hospital Care Structures (Hospotal Structure Act - KHSG)]. Bundesgesetzblatt Teil I. 2015;51:2229–53.

[12] Bundesministerium der Justiz und für Verbraucherschutz [Federal Ministry of Justice and Consumer Protection], editor. Gesetz zur Stärkung der Versorgung in der gesetzlichen Krankenversicherung (GKV-Versorgungsstärkungsgesetz) [Act to Strengthen Provision in the Statutory Health Insurance (GKV Healthcare Strengthening Act)]. Bundesgesetzblatt Teil I. 2015;30:1211–44.

[13] Gesetzesentwurf zur Reform der Notfallversorgung [Draft Law for the Reform of Emergency Care] [Internet]. Nov 17. 2025. https://www.bundesgesundheitsministerium.de/fileadmin/Dateien/3_Downloads/Gesetze_und_Verordnungen/GuV/N/RefE_Notfallreform.pdf. Accessed 9 Dec 2025.

[14] Auld BC, Abell B, Venugopal PS, McPhail S. Geographical challenges and inequity of healthcare access for high-risk paediatric heart disease. Int J Equity Health. 2023;22:229. 10.1186/s12939-023-02040-z. 37915092 10.1186/s12939-023-02040-z PMC10619221

[15] Woo JL, Anderson BR, Gruenstein D, Conti R, Chua K-P. Minimum Travel Distance Among Publicly Insured Infants with Severe Congenital Heart Disease: Potential Impact of In-state Restrictions. Pediatr Cardiol. 2019;40:1599–608. 10.1007/s00246-019-02193-1. 31463514 10.1007/s00246-019-02193-1 PMC6851488

[16] Ouma PO, Malla L, Wachira BW, Kiarie H, Mumo J, Snow RW, et al. Geospatial mapping of timely access to inpatient neonatal care and its relationship to neonatal mortality in Kenya. PLoS Glob Public Health. 2022;2:e0000216. 10.1371/journal.pgph.0000216. 36962323 10.1371/journal.pgph.0000216 PMC10021833

[17] Delmelle EM, Cassell CH, Dony C, Radcliff E, Tanner JP, Siffel C, et al. Modeling travel impedance to medical care for children with birth defects using Geographic Information Systems. Birth Defects Research Part A: Clinical and Molecular Teratology. 2013;97:673–84. 10.1002/bdra.23168. 23996978 10.1002/bdra.23168 PMC4507419

[18] Bai L, Tao Z, Cheng Y, Feng L, Wang S. Delineating hierarchical obstetric hospital service areas using the Huff model based on medical records. Applied Geography. 2023;153:102903. 10.1016/j.apgeog.2023.102903.

[19] Fülöp G, Kopetsch T, Schöpe P. Catchment areas of medical practices and the role played by geographical distance in the patient’s choice of doctor. Ann Reg Sci. 2011;46:691–706. 10.1007/s00168-009-0347-y.

[20] Latruwe T, Van Der Wee M, Vanleenhove P, Michielsen K, Verbrugge S, Colle D. Improving inpatient and daycare admission estimates with gravity models. Health Services and Outcomes Research Methodology. 2023;23:452–67. 10.1007/s10742-022-00298-4. 10.1186/s12874-023-02033-0 PMC10540423 37773104

[21] Jia P, Xierali IM, Wang F. Evaluating and re-demarcating the hospital service areas in Florida. Appl Geogr. 2015;60:248–53. 10.1016/j.apgeog.2014.10.008.

[22] Wieland T. Modellgestützte Verfahren und „big (spatial) data“ in der regionalen Versorgungsforschung II [Model-based methods and “big (spatial) data” in regional health services research II]. Monit Versorgungsforsch. 2018;11:59–64. 10.24945/MVF.03.18.1866-0533.2083.

[23] Stacherl B, Sauzet O. Gravity models for potential spatial healthcare access measurement: a systematic methodological review. Int J Health Geogr. 2023;22:34. 10.1186/s12942-023-00358-z. 38041129 10.1186/s12942-023-00358-z PMC10693160

[24] Huff DL. Determination of intra-urban retail trade areas [Internet]. Los Angeles: Real Estate Research Program, Graduate Schools of Business Administration, University of California; 1962 [cited 2025 Dec 9]. https://catalog.hathitrust.org/Record/010656400. Accessed 9 Dec 2025.

[25] Federal Institute for Research on Building, Urban Affairs and Spatial Development (BBSR). Referenztabellen zur Raumgliederung des BBSR [Internet]. 2022 [cited 2025 Mar 25]. https://www.bbsr.bund.de/BBSR/DE/forschung/raumbeobachtung/Raumabgrenzungen/downloads/download-referenzen.html. Accessed 25 Mar 2025.

[26] Bayerisches Staatsministerium für Gesundheit, Pflege und Prävention [Bavarian State Ministry of Health, Care and Prevention]. Krankenhausplan des Freistaats Bayern [Hospital Plan of the Free State of Bavaria] [Internet]. 2025 Jan. Report No.: 50. https://www.stmgp.bayern.de/wp-content/uploads/2025/02/bayerischer-krankenhausplan-2025.pdf. Accessed 9 Dec 2025.

[27] Bundesministerium für Gesundheit [Federal Ministry of Health]. Bundes-Klinik-Atlas [Federal Hospital Atlas] [Internet]. 2025 [cited 2025 Dec 9]. https://bundes-klinik-atlas.de/. Accessed 9 Dec 2025.

[28] Referenzbericht Klinik Hallerwiese/Cnopfsche Kinderklinik [Quality Report Klinik Hallerwiese/Cnopf Children’s Hospital] [Internet]. 2024. https://www.klinik-hallerwiese.de/fileadmin/user_upload/beide_Kliniken/pdf/Qualitaet/2023_QB_der_Krankenhaeuser__Nuernberg.pdf. Accessed 9 Dec 2025.

[29] Referenzbericht Universitätsklinikum Regensburg [Quality Report Regensburg University Hospital] [Internet]. 2025. https://navigatoren.aok.de/etl/public/sqb/260930608-771402000-2023.pdf. Accessed 9 Dec 2025.

[30] Referenzbericht Klinikum rechts der Isar der Technischen Universität München [Quality Report University Hospital rechts der Isar of the Technical University of Munich] [Internet]. 2025. https://mri.tum.de/sites/default/files/2025-02/qualitatsbericht_2023_stammgelande.pdf. Accessed 9 Dec 2025.

[31] Referenzbericht LMU. Klinikum - Campus Großhadern [Quality Report LMU University Hospital - Großhadern Campus] [Internet]. 2025. https://cdn.lmu-klinikum.de/b1ceb41fa5db34c9/6e3bef3078da/Standortbericht-GH-2023.pdf. Accessed 9 Dec 2025.

[32] Referenzbericht Universitätsklinikum Augsburg - Medizincampus [Quality Report Augsburg University Hospital - Medical Campus] [Internet]. 2025. https://www.uk-augsburg.de/fileadmin/Daten/Unternehmen/Bereiche_und_Stabstellen/QM/260970015-772997000-2023-xml__1_.pdf. Accessed 9 Dec 2025.

[33] Referenzbericht Universitätsklinikum Würzburg [Quality Report Würzburg University Hospital] [Internet]. 2025. https://www.ukw.de/fileadmin/uk/qm/Qualit%C3%A4tsbericht_2023_UKW.pdf. Accessed 9 Dec 2025.

[34] Referenzbericht Universitätsklinikum Erlangen [Quality Report Erlangen University Hospital] [Internet]. 2025. https://www.uk-erlangen.de/fileadmin/dateien/content_pool_dateien/UKER_qm-bericht.pdf. Accessed 9 Dec 2025.

[35] Deutsche Krebsgesellschaft e.V. [German Cancer Society (GCS)]. Zertifizierung von Krebszentren [Certification of Cancer Centers] [Internet]. 2024 [cited 2025 Dec 9]. https://www.krebsgesellschaft.de/unsere-themen/zertifizierung. Accessed 9 Dec 2025.

[36] Jia P. Developing a flow-based spatial algorithm to delineate hospital service areas. Appl Geogr. 2016;75:137–43. 10.1016/j.apgeog.2016.08.008.

[37] Jia P, Wang F, Xierali IM. Delineating hierarchical hospital service areas in Florida. Geogr Rev. 2017;107:608–23. 10.1111/j.1931-0846.2016.12207.x.

[38] Kowalski C, Graeven U, von Kalle C, Lang H, Beckmann MW, Blohmer J-U, et al. Shifting cancer care towards multidisciplinarity: the cancer center certification program of the German Cancer Society. BMC Cancer. 2017;17:850. 10.1186/s12885-017-3824-1. 29241445 10.1186/s12885-017-3824-1 PMC5731059

[39] Heidelberg Institute for Geoinformation Technology (HeiGIT). Openrouteservice [Internet]. 2025 [cited 2025 Dec 9]. https://openrouteservice.org/. Accessed 9 Dec 2025.

[40] OpenStreetMap Wiki contributors. Routing [Internet]. 2026 [cited 2026 Apr 13]. https://wiki.openstreetmap.org/wiki/Routing. Accessed 13 Apr 2026.

[41] Neis P, Zielstra D, Zipf A. The Street Network Evolution of Crowdsourced Maps: OpenStreetMap in Germany 2007–2011. Future Internet. 2011;4:1–21. 10.3390/fi4010001.

[42] Franzini M, Annovazzi-Lodi L, Casella V. Assessment of the Completeness of OpenStreetMap and Google Maps for the Province of Pavia (Italy): Proc 6th Int Conf Geogr Inf Syst Theory Appl Manag [Internet]. Prague, Czech Republic: SCITEPRESS - Science and Technology Publications; 2020 [cited 2026 Apr 13]. pp. 270–7. 10.5220/0009564302700277

[43] Huff DL. A probabilistic analysis of shopping center trade areas. Land Econ. 1963;39:81–90. 10.2307/3144521.

[44] Huff DL. Defining and estimating a trading area. J Mark. 1964;28:34–8. 10.1177/002224296402800307.

[45] Bauer J, Groneberg DA. Measuring spatial accessibility of health care providers – Introduction of a variable distance decay function within the floating catchment area (FCA) method. PLoS One. 2016;11:e0159148. 10.1371/journal.pone.0159148. 27391649 10.1371/journal.pone.0159148 PMC4938577

[46] Subal J, Paal P, Krisp JM. Quantifying spatial accessibility of general practitioners by applying a modified huff three-step floating catchment area (MH3SFCA) method. Int J Health Geogr. 2021;20:9. 10.1186/s12942-021-00263-3. 33596931 10.1186/s12942-021-00263-3 PMC7888693

[47] Wieland T. Raum- und standortökonomische Optimierungsmodelle in Open-Source-Umgebungen – Implementation und Anwendungsmöglichkeiten im Kontext der Einzelhandels- und Versorgungsforschung [Spatial and location-economic optimization models in open-source environments – Implementation and application possibilities in the context of retail and health services research]. Vienna, Austria: Selbstverlag des Vereins CORP - Compentence Center of Urban and Regional Planning; 2017. pp. 463–73.

[48] Fotheringham AS. Spatial competition and agglomeration in urban modelling. Environ Plan A Econ Space. 1985;17:213–30. 10.1068/a170213.

[49] Wieland T. Market Area Analysis for Retail and Service Locations with MCI. R J. 2017;9:298–323. 10.32614/RJ-2017-020.

[50] Orpana T, Lampinen J. Building Spatial Choice Models from Aggregate Data. J Reg Sci. 2003;43:319–48. 10.1111/1467-9787.00301.

[51] Nakanishi M, Cooper LG. Parameter Estimation for a Multiplicative Competitive Interaction Model: Least Squares Approach. J Mark Res. 1974;11:303. 10.2307/3151146.

[52] Huff DL, McCallum BM. Calibrating Huff Model Using ArcGIS Business Analyst. Redlands, CA: ESRI; 2008.

[53] Güßefeldt J. Zur Modellierung von räumlichen Kaufkraftströmen in unvollkommenen Märkten [On the modeling of spatial purchasing power flows in imperfect markets]. Erdkunde. 2002;56:351–70. 10.3112/erdkunde.2002.04.02.

[54] De Beule M, Van den Poel D, Van de Weghe N. An extended Huff-model for robustly benchmarking and predicting retail network performance. Appl Geogr. 2014;46:80–9. 10.1016/j.apgeog.2013.09.026.

[55] Li Y, Liu L. Assessing the impact of retail location on store performance: a comparison of wal-mart and kmart stores in cincinnati. Appl Geogr. 2012;32:591–600. 10.1016/j.apgeog.2011.07.006.

[56] Lee A, Cheung YSL, Joynt GM, Leung CCH, Wong W‐T, Gomersall CD. Are high nurse workload/staffing ratios associated with decreased survival in critically ill patients? A cohort study. Ann Intensive Care. 2017;7:46. 10.1186/s13613-017-0269-2. 28466462 10.1186/s13613-017-0269-2 PMC5413463

[57] Chau JPC, Lo SHS, Choi KC, Tong DWK, Kwok AML, Butt L, et al. Associations between nurse-to‐patient ratio, nurse educational level, and nurse‐sensitive patient outcomes: a 12‐month prospective observational study. Int Nurs Rev. 2025;72:e13091. 10.1111/inr.13091. 39784291 10.1111/inr.13091 PMC11714340

[58] Python Software Foundation. Python [Internet]. 2025 [cited 2025 Dec 9]. https://www.python.org/downloads/release/python-31211/. Accessed 9 Dec 2025.

[59] Wieland T, huff: Market Area Analysis in Python. [Computer software]. Freiburg, Germany; 2025. https://pypi.org/project/huff/.

[60] Byrd RH, Gilbert JC, Nocedal J. A trust region method based on interior point techniques for nonlinear programming. Math Program. 2000;89:149–85. 10.1007/PL00011391.

[61] Virtanen P, Gommers R, Oliphant TE, Haberland M, Reddy T, Cournapeau D, et al. SciPy 1.0: fundamental algorithms for scientific computing in Python. Nat Methods. 2020;17:261–72. 10.1038/s41592-019-0686-2. 32015543 10.1038/s41592-019-0686-2 PMC7056644

[62] Isfort M, Klie T. Monitoring Pflegepersonalbedarf Bayern 2023 [Monitoring Nursing Staff Requirements Bavaria 2023] [Internet]., Munich G. 2023 p. 248. https://www.vdpb-bayern.de/wp-content/uploads/2024/01/240115-Monitoring-Pflegepersonalbedarf-Bayern-2023-VdPB.pdf. Accessed 9 Dec 2025.

[63] Wohngeld- und Mietbericht [Housing Benefit and Rent Report] [Internet]. 2023 May. https://www.bfw-newsroom.de/wp-content/uploads/2023/07/Wohngeld_und_Mietenbericht_2021_22.pdf. Accessed 18 Dec 2025.

[64] Sander M, Loos S, Temizdemir E. Analyse der Situation der Pflege und Geburtshilfe (Hebammen) in den Münchner Krankenhäusern [Analysis of the situation of nursing and obstetrics (midwives) in Munich hospitals]. Berlin, Germany; p. 179.

[65] Fotheringham AS, Wong DWS. The modifiable areal unit problem in multivariate statistical analysis. Environ Plan A. 1991;23:1025–44. 10.1068/a231025.

[66] Delmelle EM, Desjardins MR, Jung P, Owusu C, Lan Y, Hohl A, et al. Uncertainty in geospatial health: Challenges and opportunities ahead. Ann Epidemiol. 2022;65:15–30. 10.1016/j.annepidem.2021.10.002. 34656750 10.1016/j.annepidem.2021.10.002

[67] Hassler J, Andersson Granberg T, Steins K, Ceccato V. Towards more realistic measures of accessibility to emergency departments in Sweden. Int J Health Geogr. 2024;23:6. 10.1186/s12942-024-00364-9. 38431597 10.1186/s12942-024-00364-9 PMC10909287

[68] Gemeinsamer Bundesausschuss (G-BA). [Federal Joint Committee]. Referenzdatenbank der Qualitätsberichte der Krankenhäuser [Internet]. [cited 2026 Apr 14]. https://qb-referenzdatenbank.g-ba.de/. Accessed 14 Apr 2026.

