# Supplementary material for: Modeling hospital catchment areas in pediatric oncology using an empirically parameterized extended Huff-model
Source: Int J Health Geogr. 2026 Jun 6;25:35. doi: 10.1186/s12942-026-00478-2 (PMC13273989; doi:10.1186/s12942-026-00478-2)
Supplement: Supplementary file 1 — Supplementary material 1 [file 12942_2026_478_MOESM1_ESM.docx]

# Supplementary


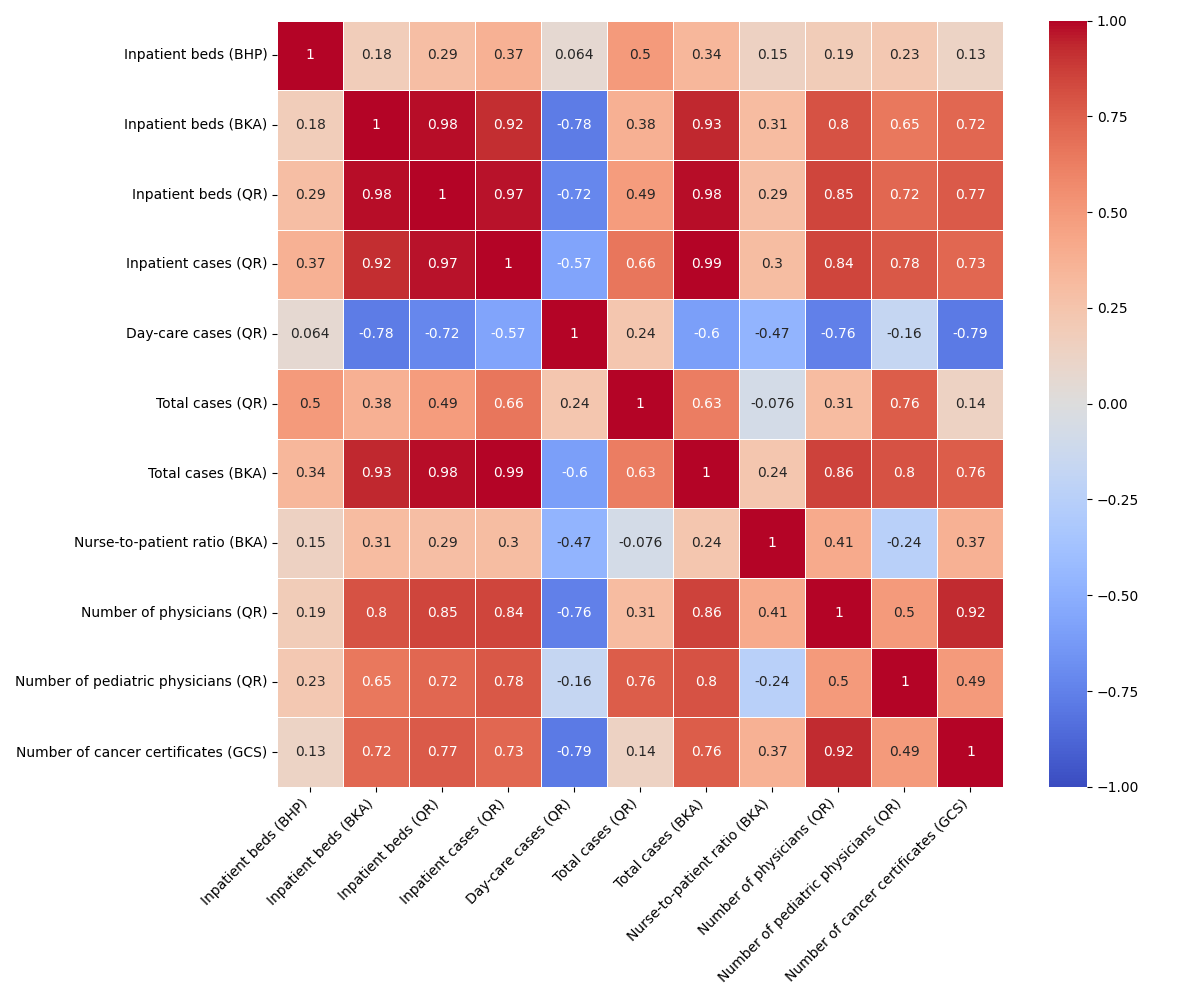


**Figure S1** Person correlation matrix. The abbreviations in parentheses indicate the respective data sources: Bavarian Hospital Plan (BHP), Bundes-Klinik-Atlas (BKA), structured quality reports of each site (QR), and the German Cancer Society (GCS). The variables *Pediatric inpatient cases* (QR), *Pediatric day-care cases* (QR), *Pediatric total cases* (QR), and *Pediatric total cases* (BKA) were excluded from the correlation analysis due to incomplete data. The variable *Oncology center accreditation* (GCS) was not included, as it is binary in nature and therefore not suitable for Pearson correlation analysis.
